# Incidence and Pattern of Graft-versus-Host Disease in Patients Undergoing Allogeneic Transplantation after Nonmyeloablative Conditioning with Total Lymphoid Irradiation and Antithymocyte Globulin

**DOI:** 10.1155/2013/414959

**Published:** 2013-04-17

**Authors:** Lauren Veltri, Michael Regier, Aaron Cumpston, Sonia Leadmon, William Tse, Michael Craig, Mehdi Hamadani

**Affiliations:** ^1^Osborn Hematopoietic Malignancy and Transplantation Program, West Virginia University, Morgantown, WV 26506, USA; ^2^Department of Biostatistics, West Virginia University, Morgantown, WV 26506, USA; ^3^Myeloma & Lymphoma Service, West Virginia University, Morgantown, WV 26506, USA

## Abstract

Nonmyeloablative (NMA) conditioning with total lymphoid irradiation and antithymocyte globulin (TLI/ATG) has been shown to protect against acute graft-versus-host disease (GVHD). We report here our institutional experience with allogeneic transplantation following NMA conditioning with TLI/ATG (*n* = 21). GVHD prophylaxis consisted of a combination of a calcineurin inhibitor and mycophenolate mofetil. Median patient age was 59 years. The median followup of surviving patients is 545 days. One patient experienced primary graft rejection. The median time to neutrophil engraftment was 18 days and platelet engraftment was 9.5 days. The cumulative incidence (CI) of grade II–IV acute GVHD at day +100 was 28.6% and 38.1% at day +180. The CI for grade III-IV acute GVHD was 28.6% at day +180. CI of chronic GVHD was 45.2% at 1 year. The CI of disease relapse was 9.5% at 1 year. The rate of nonrelapse mortality (NRM) was 0% at day +100 and only 9.5% at 1 year. The overall and progression free survival at 1 year was 81% and 80.4%, respectively. Our limited, retrospective data show encouraging relapse and NRM rates with TLI/ATG-based NMA conditioning, but with higher than previously reported rates of acute and chronic GVHD, underscoring the need for novel strategies designed to effectively prevent GVHD.

## 1. Introduction

Allogeneic hematopoietic cell transplantation (HCT) is a potentially curative modality for a variety of hematological malignancies [1, 2]. However, the high rates of procedure-related toxicities and nonrelapse mortality (NRM) have limited the applicability of HCT following conventional myeloablative conditioning regimens, to select cohort of younger patients with few or no comorbidities [3–8]. Hematological malignancies disproportionately affect the elderly [3]. From 2010 to 2030, the percentage of all cancers diagnosed in older adults in the United States will increase from 61% to 70% [9]. This change in demographics places an even greater emphasis on creating less toxic conditioning regimens, suitable for elderly or less fit individuals. The introduction of nonmyeloablative (NMA) and reduced-intensity conditioning (RIC) regimens has extended the use of allogeneic HCT to patients otherwise ineligible for conventional myeloablative HCT due to age or comorbidities [3, 10]. However, even with the decline in transplant-related toxicities with the introduction of NMA regimens, graft-versus-host disease (GVHD) remains a major cause of morbidity and mortality [11–16]. 

NMA conditioning with total lymphoid irradiation (TLI) and antithymocyte globulin (ATG) has been shown in previous studies to protect against acute GVHD, while preserving graft-versus-malignancy (GVM) effects in both murine models and in the clinical realm [17–23]. NMA conditioning with TLI/ATG alters the host immune system to favor natural killer T cells, that suppress GVHD through polarization of donor conventional T cells towards secretion of noninflammatory cytokines (e.g., interleukin 4) and by promoting expansion of donor regulatory (CD4^+^CD25^+^FoxP3^+^) T cells [19, 20, 22]. In the clinical setting, TLI and ATG-based conditioning has demonstrated low rates of NRM and acute GVHD (<10% at day +100) [17, 19, 21]. Data on the incidence of late onset, classical acute GVHD in patients conditioned with TLI and ATG, beyond day +100, are not available. We describe here our institutional experience with allogeneic HCT following NMA conditioning with TLI/ATG and report the incidence and pattern of early acute, late acute, and chronic GVHD. 

## 2. Patients and Methods

### 2.1. Patient Population

Patients with hematological malignancies or bone marrow failure syndromes undergoing allogeneic HCT following NMA conditioning with TLI/ATG, between November 2007 and March 2012 at our Blood and Marrow Transplantation Program were included. The criteria used to offer TLI/ATG conditioning at our institution include presence of adverse-risk features (that preclude the use of higher-intensity conditioning regimens) defined by the presence of at least one of the following features: (i) age ≥60-years; (ii) Karnofsky performance score (KPS) ≤70; (iii) hematopoietic cell transplantation-comorbidity index (HCT-CI) ≥4 [24]; (iv) baseline diagnosis of follicular lymphoma, or chronic lymphocytic leukemia; and (v) prior history of autologous transplantation. This study was approved by the Institutional Review Board and Clinical Scientific Review Committee.

### 2.2. Conditioning Regimen

**Figure 1:**
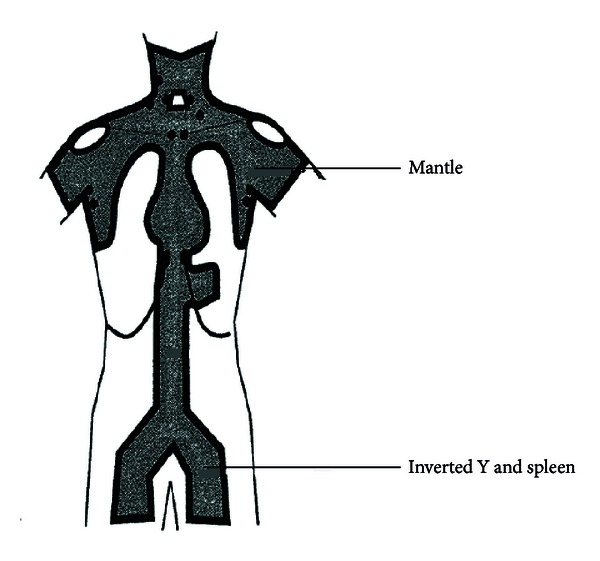
Lymph node regions included in total lymphoid irradiation.

TLI/ATG conditioning regimen was delivered, as described previously [17, 19]. Briefly, neck, chest, abdomen, and pelvic computed tomography scans were obtained in all subjects and target volumes were outlined. The irradiation (i.e., clinical target volume) consisted of mantle field, a subdiaphragmatic field that included an inverted-Y, and splenic ports encompassing all major lymphoid organs (including the thymus, spleen, and lymph nodes) (Figure 1). Thoracic and abdominal organs at risk were contoured. TLI was administered from a 15 MeV linear accelerator (photon beam), ten times in 80 cGy fractions (total dose 800 cGy) on day 11 through day 7 and day 4 through day 1, using a 3D-conformal technique. One dose per day was given on days 11 through 7, and days 4 through 2. Two doses four hours apart were given on day 1. Field junctions were required at the top of the spleen, and a second junction occasionally required, due to field size constraints, at the top of the pelvis. Isodose contours included the lymphoid regions and spleen at the 95% isodose levels. At field junctions, the 50% decrement lines of adjoining fields intersected at the midplane. Evenly weighted anterior-posterior and posterior-anterior fields were treated using between 6 and 15 MV photons from a linear accelerator. All fields were treated in each treatment session. ATG (Thymoglobulin, Genzyme, Cambridge, MA) was administered from day 11 through 7 at the dose of 1.5 mg/kg/day. Hematopoietic stem cell infusion was performed on day 0.

### 2.3. HLA Typing and Chimerism Analysis

High-resolution typing for human leukocyte antigen (HLA) class I (HLA-A,-B,-C) and class-II (HLA-DRB1, -DQB1) alleles was performed by polymerase chain reaction-sequence specific primary (PCR-SSP) amplification, as described previously [25]. To assess donor-cell chimerism, peripheral blood samples were collected before transplantation to identify PCR-short tandem repeat informative fragments for each donor/recipient pair. After transplantation chimerism analysis was performed on days +30, +100, +180, and +365. Complete donor chimerism was defined as presence of ≥95% donor cells. Primary graft rejection was defined as failure to establish hematopoietic reconstitution of donor-origin after allografting, while secondary graft rejection was defined as confirmed loss of donor cells after initial donor-origin hematopoiesis. Secondary graft failure was defined as absolute neutrophil count (ANC) <0.5 × 10^9^/L, after initial neutrophil recovery after HCT.

### 2.4. GVHD Prophylaxis

Patients undergoing matched sibling donor (MSD) HCT received GVHD prophylaxis with oral cyclosporine (6.25 mg/kg twice a day; starting on day 3) and mycophenolate mofetil (MMF; 15 mg/kg twice a day; starting on the evening of day 0, after stem cell infusion). Patients undergoing an unrelated donor (URD) HCT received tacrolimus (0.015 mg/kg/day starting at day 3) and MMF. Blood levels of cyclosporine and tacrolimus were monitored twice weekly. In the absence of acute GVHD or disease relapse, among recipients of MSD allografts, cyclosporine was tapered to discontinuation from day 56 to day 180, and MMF was stopped on day 28. Among recipients of URD transplants, tacrolimus was tapered to discontinuation between day 100 and day 180, and MMF was tapered to discontinuation from day 42 to day 96.

### 2.5. Transplantation Procedure and Supportive Care

All patients were treated in HEPA-filtered rooms and received fungal (fluconazole), herpes zoster/herpes simplex (acyclovir or valacyclovir), bacterial (ciprofloxacin or levofloxacin), and *Pneumocystis jiroveci* prophylaxis (trimethoprim/sulfamethoxazole or dapsone). Monitoring for cytomegalovirus (CMV) and Epstein-Barr virus (EBV) reactivation by quantitative PCR was conducted. Preemptive ganciclovir or valganciclovir were administered to patients with CMV reactivation (defined as ≥4000 copies/mL, reconfirmed within 24 hours from initial detection); preemptive single intravenous dose of rituximab (375 mg/m^2^) was administered to patients with evidence of EBV replication (defined as ≥4000 copies/mL, reconfirmed within 24 hours from initial detection). Urine and/or serum BK-virus PCR was obtained in all suspected cases of hemorrhagic cystitis. The time of neutrophil engraftment was considered the first of three successive days with ANC ≥0.5 × 10^9^/L after posttransplantation nadir. The time of platelet engraftment was considered the first of seven consecutive days with platelet count 20 × 10^9^/L or higher, in the absence of platelet transfusion for preceding seven days. 

### 2.6. GVHD Assessment and Treatment

Patients achieving neutrophil engraftment were evaluable for acute GVHD. Acute GVHD was graded using standard criteria [26]. All potential cases of skin and gastrointestinal acute GVHD were confirmed on histological examination of representative biopsy specimens. Cases of liver only acute GVHD were also confirmed by either a transjugular or transcutaneous core needle biopsies. Patients were evaluable for chronic GVHD if engraftment occurred and the patient survived for 100 days after transplantation. The diagnoses of limited and extensive chronic GVHD were made as previously described [27–29], while classification of chronic GVHD into mild, moderate, and severe subtypes was performed by using the National Institutes of Health Consensus Development Project Criteria [30]. Corticosteroids comprised the first-line therapy of acute (grade II–IV) and extensive chronic GVHD. Second-line treatment was at the discretion of treating physicians. 

### 2.7. Statistical Analysis

Descriptive statistics were calculated for the baseline variables of the whole cohort. Overall survival (OS) and progression free survival (PFS) were estimated using the Kaplan-Meier method. OS was defined as the time from transplant to death from any cause, and surviving patients were censored at last followup. PFS from transplantation was calculated using death and disease progression and/or relapse as events. NRM was defined as death from any cause other than disease progression or relapse. Cumulative incidence was estimated for NRM and relapse risk, with relapse as a competing risk for the former and death in remission for the latter [31]. Gray's test was used to assess the difference between various subgroups for NRM and relapse rate. The probability of developing acute GVHD or chronic GVHD was depicted by calculating the cumulative incidence with relapse and death without relapse or acute GVHD or chronic GVHD as competing risks [14, 31]. Variables associated with acute and chronic GVHD, NRM, and relapse in the presence of a competing risk were tested individually using competing risk regression [32]. All *P* values are two sided. Analyses were run using the statistical software R (http://www.r-project.org/).

## 3. Results

### 3.1. Patient Characteristics

The baseline characteristics of 21 patients included in this study are shown in Table 1. The median age at baseline was 59 years (range 26–72), with nine patients ≥60 years of age at transplantation. These patients received a median of 2 prior lines of therapies (range 1–6). At the time of transplantation 3 patients had chemotherapy refractory disease. Eight patients had an HCT-CI of ≥3 at baseline. All except one patient received filgrastim-mobilized peripheral blood allografts. Five patients (23.8%) underwent an MSD HCT. One patient undergoing an URD allogeneic transplant had a one allele-level mismatch at HLA-B with the donor. Twelve patients had KPS of <90 at baseline. 

### 3.2. Engraftment and Chimerism

Twenty patients in the study successfully engrafted with donor cells. One patient experienced primary graft rejection and subsequently had autologous recovery (day +30 chimerism = 100% recipient cells). The median time to neutrophil engraftment was 18 days (range 9–82 days) and platelet engraftment was 9.5 days (range 6–24 days). Post HCT the ANC did not decrease below 500/*μ*L in 8 patients. Four patients had secondary graft failure, which resolved with growth factor support. Median donor chimerism on days +30, +100, +180, and +365 was 92.5% (range 0–100%), 94% (range 54–100%), 100% (range 56–100%), and 100% (range 88–100%), respectively. Donor lymphocyte infusions were administered in two patients (indication: decreasing donor chimerism and relapse acute myeloid leukemia).

### 3.3. GVHD

**Figure 2:**
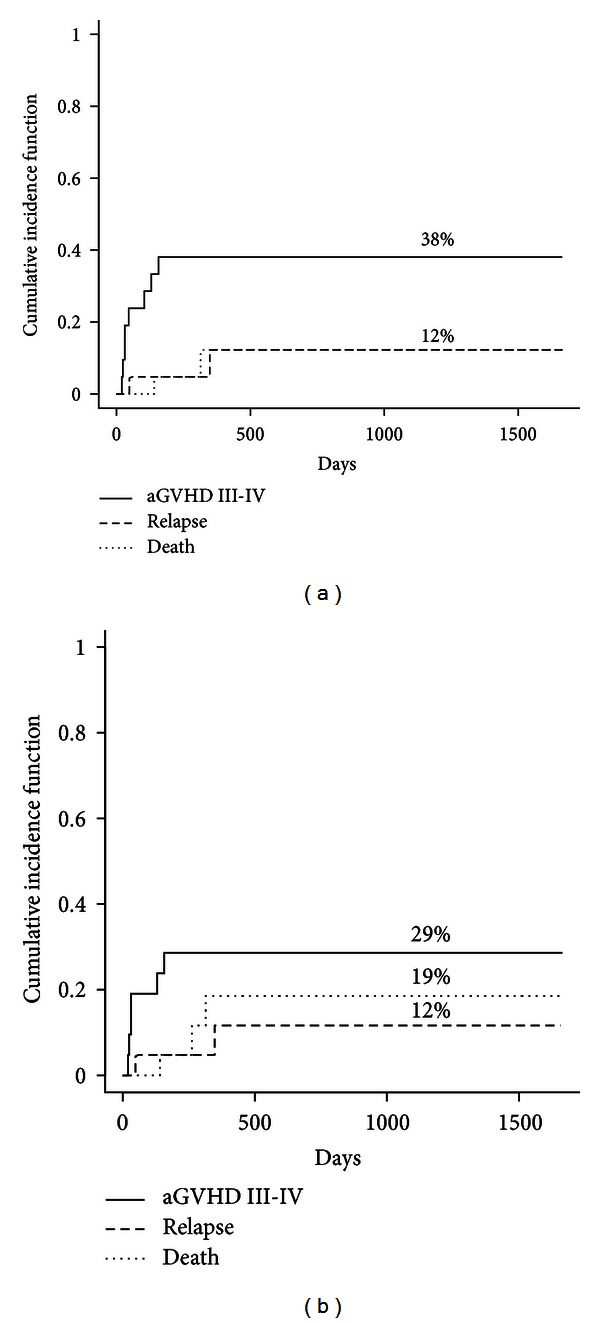
Cumulative incidence of grade II–IV (a) and grade III-IV (b) acute GVHD after transplantation (solid curves: acute GVHD, interrupted curves: competing events).

Twenty patients were evaluable for acute or chronic GVHD. The median time to tapering patients completely off immunosuppression was 218 days (range 78–458 days). Median time to onset of acute GVHD was 38 days (range 20–157). All acute GVHD cases were biopsy proven. The cumulative incidence of grade II–IV acute GVHD while accounting for competing events at day +100 and day +180 was 28.6% (95% CI = 1.2–62.9) (*n* = 5) and 38.1% (95% CI = 19.0–57.1) (*n* = 8), respectively (Figure 2(a)). The cumulative incidence for grade III-IV acute GVHD at day +100 and day +180 was 19% (95% CI = 0.1–69.1) (*n* = 3) and 28.6% (95% CI = 5.0–59.3) (*n* = 5), respectively (Figure 2(b)). The rate of acute GVHD was 20% (*n* = 1) and 46.6% (*n* = 7) in the recipient of MSD and URD transplants, respectively. When grouping the events by donor source (MSD versus URD), Gray's test indicated that there was no statistically significant difference between the two donor groups for grade II–IV acute GVHD (*P* = 0.429).

**Figure 3:**
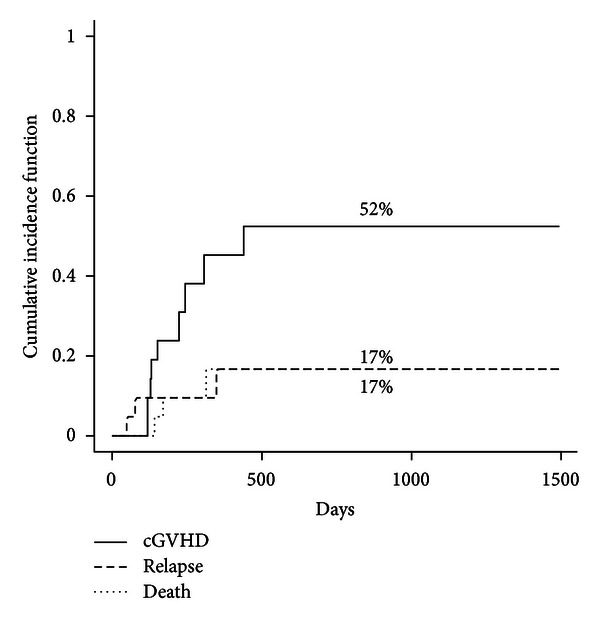
Cumulative incidence of chronic GVHD after transplantation (solid curves: chronic GVHD, interrupted curves: competing events).

Median time to the onset of chronic GVHD was 151 days (range 118–439). The cumulative of chronic GVHD was 45.2% (95% CI = 28.9–60.3) at 1 year (Figure 3), while the cumulative incidence of limited and extensive chronic GVHD was 18.6% (*n* = 3) and 25.9% (*n* = 5), respectively, at 1 year. The cumulative incidence of mild-moderate (*n* = 5) and severe (*n* = 3) chronic GVHD was 25.9% and 18.6%, respectively at 1  year. When grouping the events by donor source, Gray's test indicated a weak statistical significance between the MSD and URD groups for chronic GVHD (*P* = 0.188). 

### 3.4. Infectious Complications

Viral reactivations were frequent. Fifteen patients (71.4%) had CMV reactivation, while 2 experienced EBV reactivation. There were no cases of posttransplant lymphoproliferative disorder. Two patients had BK-virus associated episodes of hemorrhagic cystitis. Eleven patients (52.4%) experienced bacterial infections in their posttransplant course. One patient developed an invasive fungal infection.

### 3.5. Nonrelapse Mortality and Relapse Rate

**Figure 4:**
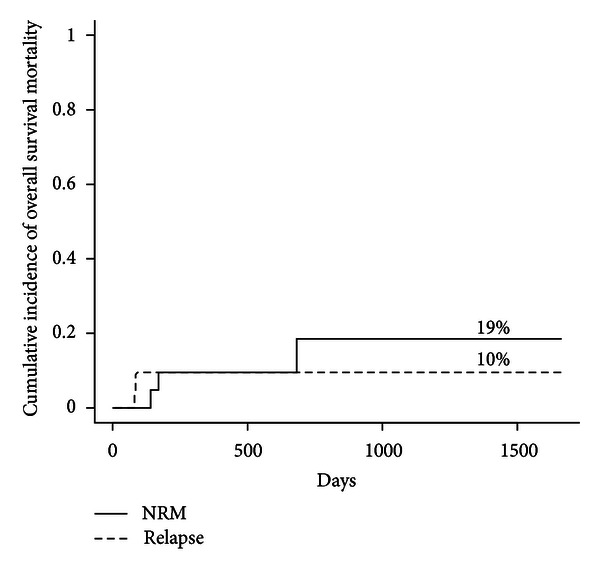
Cumulative incidence of NRM (solid curve) and disease relapse (interrupted curve), after transplantation.

The cumulative of disease relapse was 9.5% (*n* = 2) at 1 year. The cumulative incidence of NRM at day +100 and 1 year was 0% and 9.5%, respectively (Figure 4). At last followup, five patients had died. Causes of death included disease relapse (*n* = 2), GVHD (*n* = 2), and motor vehicle accident (*n* = 1). When grouping the study population by donor source (MSD versus URD), Gray's test indicated that there was no statistically significant difference between the two donor groups for either NRM or relapse rates (*P* = 0.995 and *P* = 0.418 resp.). 

### 3.6. Overall and Progression Free Survival

**Figure 5:**
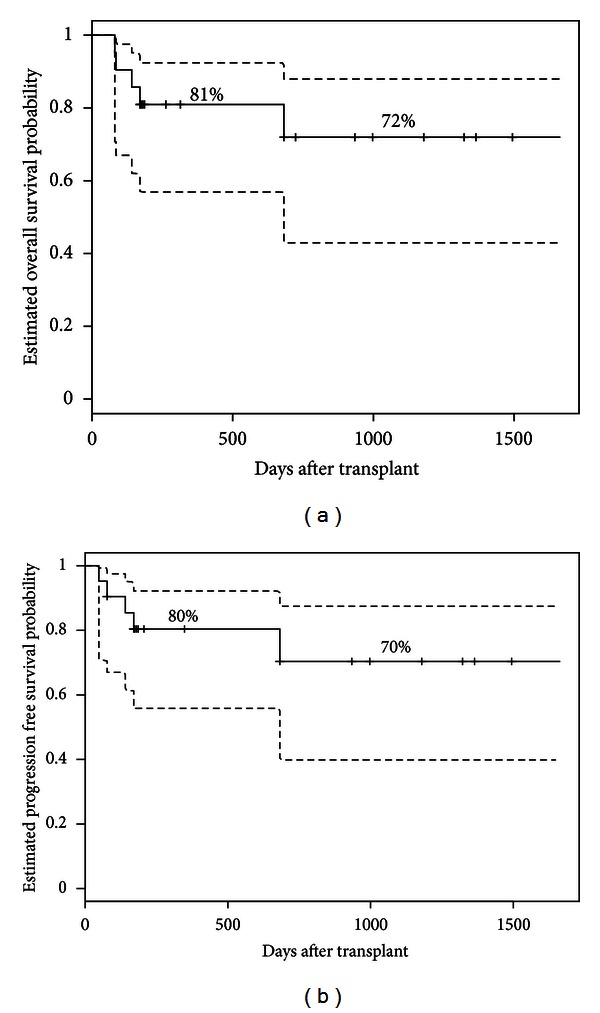
Kaplan-Meier estimates of overall survival (a) and progression free survival (b) after transplantation (interrupted curves = 95% confidence intervals).

The median follow-up days of surviving patients are 545 days (range 174–1664). At the last followup, 16 patients were alive. The 1-year and 3-year probabilities of OS were 81% and 72%, respectively (Figure 5(a)). The respective figures for PFS are 80.4% and 70.4% (Figure 5(b)). There was no significant impact of donor source, patient age, or donor-cell chimerism on OS or PFS.

## 4. Discussion

Lower-intensity conditioning regimens (including the so-called RIC and NMA regimens) have extended the applicability of allogeneic HCT to elderly patients and those with comorbidities. In the current study, we report our institutional experience (extracted from a prospectively maintained database) with TLI/ATG-based NMA conditioning and make several interesting observations. First, despite including mostly elderly patients or those with significant comorbidities, TLI/ATG conditioning was associated with low NRM. Second, this truly NMA regimen was able to provide durable disease control, in this cohort of predominantly chemosensitive patients. Third, unlike prior reports, in our study, the rates of acute GVHD and chronic GVHD were high. Fourth, viral reactivations and infectious complications were frequent, but manageable. 

In the updated experience from the Stanford group, the rates of grade II–IV acute GVHD at day +100 following TLI/ATG conditioning were 2% and 10% in patients receiving MSD and URD grafts, respectively [19]. Rates of late onset, classical acute GVHD were, however, not reported. Messina et al. in an Italian study reported acute GVHD rate of approximately 13% [21]. In contrast to these prior reports, our data demonstrate much higher rates of acute GVHD. Data regarding the incidence of late onset, classical acute GVHD following TLI/ATG conditioning are limited. Our study shows that late onset acute GVHD is not infrequent after TLI/ATG, with cumulative incidence of acute GVHD increasing from 28.6% (*n* = 5) at day +100 to 38.1% (*n* = 8) at day +180. The reasons for the higher rates of acute GVHD in our study compared to prior reports are not readily apparent. The proportion of URDs, HLA-mismatched recipients, and GVHD prophylaxis employed in our report is comparable to prior reports of TLI/ATG [17, 18, 21]. Interstudy variations in the observed incidence of acute GVHD have previously been shown by others to be a reflection of how aggressively diagnostic endoscopy is pursued to assess the etiology of gastrointestinal disturbances [15, 33]. Our institutional practice of aggressively obtaining biopsy confirmation in all presentations suspicious for acute GVHD can be a reason for observed higher rates in our study. It is however important to note that all acute GVHD cases in our study had biopsy confirmation and that steroid refractory acute GVHD was the cause of death for two patients in our study, and a third patient who relapsed after transplant also had steroid refractory acute GVHD at the time of death. We substituted tacrolimus for cyclosporine in patients undergoing URD transplantation. This is unlikely to be responsible for our observed higher GVHD rates, as randomized data suggest improved efficacy of tacrolimus for GVHD prophylaxis compared to cyclosporine in URD transplantation [34]. We cannot discount the possibility that our data might merely be a reflection of a “center volume effect.” The cumulative incidence of chronic GVHD (45.2% at 1 year) is also higher than previous TLI/ATG reports (26%–35%) [19, 21], but comparable to rates expected following non-TLI/ATG containing RIC or NMA regimens [12, 14, 34, 35]. 

The rate of CMV reactivation in our experience was high (71.4%), likely due to ATG mediated T-cell depletion. In the report by Messina et al. [21], CMV reactivation was observed in 44% of the patients, while reactivation rates were not reported by Kohrt et al. [19]. The higher incidence of GVHD in our series and associated immunosuppressive therapy could have contributed to frequent CMV reactivations, as previously reported by others [36, 37]. In fact, rate of CMV reactivation in our study in patients with acute GVHD (87.5%; 7 of 8 patients with acute GVHD reactivated CMV) was higher compared to the rate in patients without acute GVHD (61.5%), albeit nonsignificantly (*P* = 0.20). The rates of CMV reactivation in patients with or without chronic GVHD in our study were 55% and 83%, respectively (*P* = 0.19). Despite the high acute GVHD rates in our study, the NRM and relapse rates remain reassuring. The rate of NRM in the Stanford experience was 3% at 1 year, whereas the Italian study reported a 1 year NRM of 9.1% [19, 21]. Despite employing a truly NMA conditioning, the low rates of disease relapse in our study are noteworthy. It is possible that these low relapse rates are partially due to augmented GVM effects associated with development of GVHD or CMV reactivation, as reported recently [38]. One patient receiving a bone marrow product in our experience had primary graft rejection, raising the possibility that bone marrow might not be an optimal graft source for TLI/ATG based allogeneic HCT. It must however be noted that graft rejection rates of up to 5% with TLI/ATG have previously been reported [18, 19]. 

In conclusion, acknowledging the limitation of our study including its retrospective nature and small sample size, it appears that NMA conditioning with TLI/ATG provides durable disease control, with low rates of disease relapse and NRM. Clinically significant acute GVHD however remains frequent and problematic, underscoring the need for continued investigations to prevent this frequent cause of transplant morbidity and mortality.

## Figures and Tables

**Table 1:** Baseline patient and disease characteristics.

Characteristics	*N* = 21
Median age; years (range)	59 (26–72)
Male gender (%)	14 (66.7)
Diagnosis (%)	
Acute myeloid leukemia	4
Chronic lymphocytic leukemia	3
Chronic myeloid leukemia (blast crisis)	2
B-cell NHL	9
T-cell NHL	2
Hemophagocytic syndrome	1
Disease relapse risk* (%)	
Standard risk	15 (71.4%)
High risk	5 (23.8%)
Unknown	1 (4.8%)
Prior autologous transplant (%)	
Yes	1 (4.8%)
No	20 (95.2%)
Donor type (%)	
Related donor	5 (23.8%)
Unrelated donor	16 (76.2%)
Sex mismatch^†^ (%)	
M → M	10 (47.6%)
M → F	3 (14.3%)
F → M	4 (19.0%)
F → F	4 (19.0%)
Degree of HLA match (%)	
10/10	20 (95.2%)
9/10	1 (4.8%)
Median KPS (range)	80 (70–100)
Median HCT-CI (range)	1 (0–5)
Cytomegalovirus status (%)	
Patient or donor +	11 (52.4%)
Both patient and donor +	5 (23.8%)
Both patient and donor negative	4 (19.0%)
Unknown	1 (4.8%)
ABO mismatched	8
Median CD34 cell dose infused (10^6^ cells/kg recipient) (range)	6.0 (1.5–11.3)

^†^Donor → Patient.

Abbreviations: HCT-CI: hematopoietic cell transplantation comorbidity index; KPS: Karnofsky performance score; NHL: non-Hodgkin lymphoma.

*Disease relapse-risk classification based on standard ASBMT criteria (available at http://www.asbmt.org/displaycommon.cfm?an=1&subarticlenbr=35/ (Last accessed February 1, 2013)).
